# What will be the future of computational biology for macromolecules in the era of AI?

**DOI:** 10.1371/journal.pcbi.1014467

**Published:** 2026-07-08

**Authors:** Arne Elofsson, Nir Ben-Tal

**Affiliations:** 1 Science for Life Laboratory and Department of Biochemistry and Biophysics, Stockholm University, Stockholm, Sweden; 2 School of Neurobiology, Biochemistry and Biophysics, The George S. Wise Faculty of Life Sciences, Tel Aviv University, Tel Aviv, Israel; Johns Hopkins University, UNITED STATES OF AMERICA

## Abstract

We have seen more progress in computational biology for macromolecules in the last five years than we experienced in the five preceding decades. Thus, it is very challenging to forecast future progress. It is possible that we have reached a plateau, and we will be stuck with similar problems as we have today. Still, it is also possible that the field will continue its rapid progress and completely transform other fields, such as biochemistry, molecular and cell biology, and medicine. It is also possible that general AI will take over, and all scientific endeavours will be conducted without human input. To be honest, we do not know what will happen, but we will highlight a few of the challenges and the most critical research questions that we face today. Hopefully, these will be resolved within the following decades, or hopefully much earlier. Looking back over the last decade, we can see that machine learning and deep learning have become significantly more popular (*T*-test residual > 2) among the papers published within our section of PlosCB. We do believe that this trend will continue; therefore, we focus on the challenges that must be overcome for it to make significant and notable contributions. The future of computational biology for macromolecules in 20 years is likely to be characterised by transformative advances in accuracy, automation, integration, and explainability, with AI playing a role in one form or another.

## Structure prediction of macromolecules

**Fig 1:**
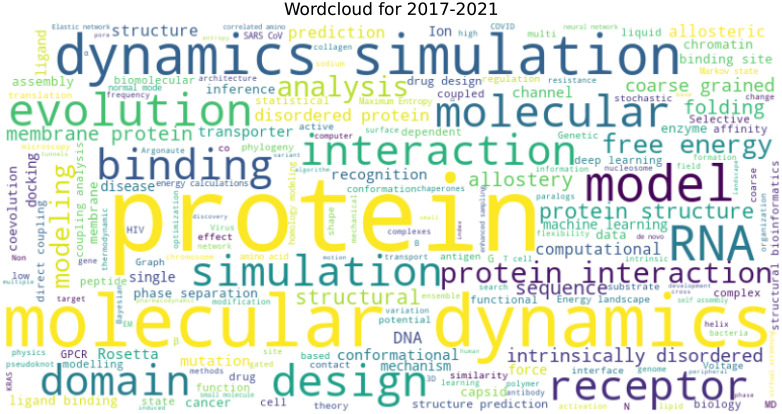
Word Clouds for papers published in our section of PlosCB in 2017–21. The only statistically significant difference between this set and the one in Fig 2 is that “machine learning” and “deep learning” have become more frequent in the latter set.

**Fig 2:**
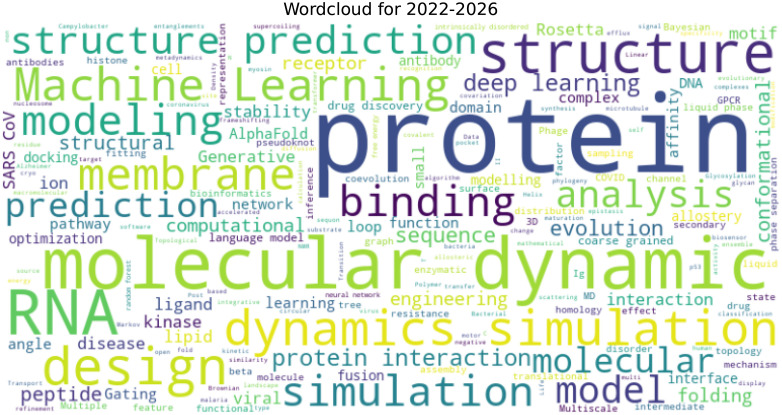
Word Clouds for papers published in our section of PlosCB in 2022–26. The only statistically significant difference between this set and the one in Fig 1 is that “machine learning” and “deep learning” have become more frequent in the latter set.

During the last decade, we can see that machine learning and deep learning have become significantly more popular (*T*-test residual > 2) among the papers published within our section of PlosCB, Figure 1 and 2. Notably, AlphaFold2 [1] revolutionised the predictions of protein structures, and later of protein interactions, in 2020 and 2021 [2]. It works astoundingly well when co-evolution can guide predictions. However, in cases where there is no co-evolution, such as antibody-antigen or host-parasite interactions, performance drops significantly. Also, predictions of (not overly stable) de novo-designed proteins (or orphan proteins) are much less accurate than for proteins belonging to large protein families.

Since the introduction of AlphaFold, several attempts have been made to extend these ideas to other molecules, including small molecules and RNA. Some of the most notable attempts are AlphaFold3 [3] and RoseTTAFold AllAtom [4], which can predict the structure of all types of molecules (except water) present in the PDB. However, it is becoming increasingly clear that when these methods extend beyond proteins (by incorporating co-evolutionary information), accurate predictions are primarily obtained for structures with similarity to those in the training set. Likely, a better way to describe the physics of the interactions between atoms is necessary when there is no co-evolutionary information. Several attempts have been made to utilise AI to describe the physics of biological macromolecules more accurately [5]. However, it remains to be shown that these methods go beyond memorisation of the training data. Alternatively, improved simulation methods, both classical molecular dynamics (MD) and ab initio quantum chemistry, might, alone or in combination with AI, ultimately enable the scientific community to predict the structures of biological macromolecules.

In this respect, it is notable that AI also boosts quantum chemistry calculations [6]. Quantum chemistry calculations are computationally demanding and are currently only feasible for small organic and inorganic molecules, rather than entire proteins or other macromolecules. The integration of AI with quantum chemistry calculations has been proposed to provide more accurate force fields for protein simulations [7]. However, these methods are still many orders of magnitude too slow for large-scale structure predictions.

A related problem, in which co-evolution provides little information, is predicting the effects of mutations [8,9]. Although many methods based on sequence and structural information have demonstrated improved accuracy in large-scale datasets, such as ProteinGym [10], it remains unclear to what extent these methods surpass the trivial observation that placing a nonpreferred amino-acid in a conserved position of a protein is detrimental [11]. Progress in this area might be necessary for the structural modelling of individual proteomes (e.g., in cancer or rare diseases), which will be crucial for predicting personalised drug responses, immunogenicity, and synthetic lethality.

Finally, it is also important to predict not only the structures of proteins and other macromolecules but also those of small molecules, i.e., ligands and/or drugs [5]. Here, the development of RoseTTAFold AllAtom [4] and AlphaFold3 [3] was followed by an explosion of programs in recent years [12–15]. In benchmarks, these methods all perform well at predicting a ligand’s location. However, it remains unclear how well these methods perform on tasks dissimilar to those in the training set. These AI-based methods also claim to perform as well as physics-based or expensive MD-based methods in predicting the binding strength of small molecules [16]

### The future

It is clear that over the next decade, AI will improve our ability to predict macromolecular structures. However, it remains to be seen whether this progress stems from a genuine understanding of the physics or merely from memorisation of the data used to train the models. Perhaps a fundamental problem that should be solvable is the accurate prediction of protein structures without relying on evolutionary information, whether in the form of an MSA or a Protein Language Model. Until that is solved, we cannot claim to have solved the protein folding problem.

## Dynamics of macromolecules

Macromolecules do not act by structure alone; they are also dynamic entities. Dynamics occur on a wide range of time scales, from picoseconds to years, and are essential for many biological functions. Classically, the most successful way to study macromolecular dynamics computationally has been to use MD simulations. However, these are limited both by size (millions of atoms) and time (milliseconds). Coarse-grained models, particularly those that use implicit water models, can accelerate simulations by one or two orders of magnitude, albeit at the cost of accuracy.

AI can be used both to improve MD simulations [7] and as a complementary method to generate the ensemble of conformations, which are otherwise collected as snapshots from MD trajectories [17]. AI has been used to learn new energy functions for amino-acid interactions. Here, the data was obtained from (time-consuming) DFT calculations and then learned by a neural network [7]. Interactions with water were still treated using the classical energy functions. The authors claimed that the new energy functions were more accurate. Several similar approaches have been used to improve the energy functions used by coarse-grained models [16,18]. This will be particularly important for understanding disordered proteins [19]. The future will tell if AI will correct and guide force fields based on learned physical priors from quantum chemistry and empirical data.

In theory, it is possible to accelerate MD simulations by predicting the structural changes from a starting conformation. In this way, much longer time-steps could be used. However, given the compactness of biological macromolecules and the importance of water, it remains to be proven if such an approach has a future. Here, one major problem is the lack of data, i.e., can MD simulations be used to generate the necessary data, and are they accurate enough for the time scales we are interested in?

Alternatively, AI methods can be used directly to generate an ensemble of structural models [7,17]. Soon after the release of AlphaFold2, it became clear that alternative conformations could sometimes be generated. This ability has been enhanced by introducing noise, either through modifications to the multiple sequence alignment (via clustering or masking) or by enabling dropouts during inference. It is now clear that most known examples of proteins with alternative conformations can be generated. Still, it remains to be proven whether the generated structures are good representatives of the ensemble.

Nevertheless, deep learning will likely enhance MD (ML-accelerated MD), enabling simulations on the microsecond-to-second timescale, which is required to model whole eukaryotic cells.

### The future

Clearly, AI methods will enable improved simulations and understanding of the dynamical processes of macromolecules. The exact methodology that will be most successful is unclear: it may be achieved by improving MD simulations (making them more accurate and/or faster) or by providing entirely novel tools to generate macromolecular ensembles. It will also be important to develop experimental methods to test and improve predictions from computational models.

## Scaling up computational methods to study macromolecules

To link molecular to organism-level behaviour, it is necessary to scale current predictions and simulation methods to much larger scales than is standard today. Ideally, models should describe entire cells (or even organisms or bacterial communities). When this is possible, structural models will link the dynamic behaviour of macromolecules to cellular phenotypes using multi-resolution simulations. Ultimately, such models will capture not only structure but function and regulation in context (e.g., enzymes within metabolic networks, RNA within condensates). Early attempts at this for small bacterial cells have shown that even obtaining the parts list (i.e., the exact number of each molecule present in a cell) is not trivial [7,17,20]. At the next order of complexity, it is also necessary to predict the spatial interactions among these molecules. Although some progress has been made in detecting all interactions in a proteome, using AlphaFold [21–23], this is far from being accurate enough. In particular, transient interactions cannot be systematically predicted today.

In addition, it has been demonstrated that even cells that appear identical are not. In simple IC50 drug test assays (where half the cells die), the process is not random. It is actually possible to predict, with some accuracy, which particular cells will die and which will survive [24,25]. This suggests that variations within cells can be used to explain biological outcomes; however, as discussed above, determining at a molecular level which variations are significant is nontrivial. The development of full-scale atomistic models of cells that are sufficiently accurate to predict phenotypic differences among the cells will be a challenge for years to come. In addition, proteomics, transcriptomics, and interactomics data will be automatically mapped onto structural models, and digital twin models of cells and tissues will integrate macromolecular networks, allowing in silico testing of interventions.

Obviously, a future goal is to perform a sufficiently detailed simulation of an entire cell (or cellular system) so that most experiments can be conducted in silico rather than experimentally. However, for different problems, both simulation time and the necessary level of detail vary by many orders of magnitude. Recently, both near-atomistic [26] and simplified [27] 4D simulations of the minimal bacterium JCVI-syn3A have been reported. The simplified model could reproduce the experimentally observed doubling time.

### The future

Scaling up the models we have today to predict and simulate macromolecules and entire cells will be an important theme in the foreseeable future. This will require not only faster computers/methods, but also increased precision in the models. Modelling of whole organisms and bacterial communities will be next on the list. With this, our understanding of biology will be based on movies that follow various processes. The film will have telescopic capabilities. Taking fertilisation as an example, scholars can start with a coarse-grained overview of sperm-egg recognition and subsequent fusion. They will be able to proceed following the same biological process at an intermediate resolution, which provides a vague indication of the macromolecules involved [28]. By repeatedly zooming in, they will be able to trace fertilisation in full atomic detail.

## Designing novel biological macromolecules

One of the significant consequences of AlphaFold2 was that an accurate computational method for testing protein designs suddenly became available to anyone. In short, if a protein (or several proteins) is predicted to fold in an expected way by AlphaFold2 (in single-sequence mode) or by ESMfold [29], this protein (or system) likely folds in this way experimentally. This capability has then been used to develop various methods for designing backbones [30,31] and sequences [32,33], which are subsequently tested/optimised before experimental validation. However, it is well known that single-sequence predictions from AlphaFold work only for very stable proteins, whereas less stable proteins are not predicted accurately. When designing a permanent binder, this is fine, as you want to have the strongest possible interaction. However, when designing other types of proteins, such as transient binders or enzymes, greater dynamical mobility is likely necessary. Although some attempts have been made to design more flexible proteins [34,35], it remains unclear which computational methods are required to achieve this consistently at scale. Again, we may need a system that can accurately describe the energetic landscape of a protein without relying on evolutionary information.

### The future

Generative models (beyond AlphaFold) will need to support the design of macromolecules with precise structural, dynamic, and functional properties. Near-instant structure prediction for proteins, nucleic acids, and complexes—even under different cellular conditions (pH, redox, crowding)—will be available.

## Final thought

In 2045, computational biology for macromolecules will likely not be a separate discipline, but rather a foundational layer of all biosciences, embedded in diagnostics, therapeutics, agriculture, and environmental biotechnology. Platforms akin to AlphaFold Server 3.0, Rosetta-X, or ESM-Y (and other protein language models) will be available through, hopefully, open-access cloud resources, making high-quality modelling universal.
